# Exposures among Pregnant Women near the World Trade Center Site on 11 September 2001

**DOI:** 10.1289/ehp.7694

**Published:** 2005-02-10

**Authors:** Mary S. Wolff, Susan L. Teitelbaum, Paul J. Lioy, Regina M. Santella, Richard Y. Wang, Robert L. Jones, Kathleen L. Caldwell, Andreas Sjödin, Wayman E. Turner, Wei Li, Panos Georgopoulos, Gertrud S. Berkowitz

**Affiliations:** ^1^Mount Sinai School of Medicine, New York, New York, USA; ^2^Environmental and Occupational Health Sciences Institute, Robert Wood Johnson Medical School–University of Medicine and Dentistry of New Jersey, Piscataway, New Jersey, USA; ^3^Mailman School of Public Health, Columbia University, New York, New York, USA; ^4^Centers for Disease Control and Prevention, Atlanta, Georgia, USA

**Keywords:** dust, exposure index, metal, PAH, particulate, PBDE, PCB, polychlorinated dibenzodioxins, polychlorinated dibenzofurans, pregnant, WTC, WTC plume

## Abstract

We have characterized environmental exposures among 187 women who were pregnant, were at or near the World Trade Center (WTC) on or soon after 11 September 2001, and are enrolled in a prospective cohort study of health effects. Exposures were assessed by estimating time spent in five zones around the WTC and by developing an exposure index (EI) based on plume reconstruction modeling. The daily reconstructed dust levels were correlated with levels of particulate matter ≤ 2.5 μm in aerodynamic diameter (PM_2.5_; *r* = 0.68) or PM_10_ (*r* = 0.73–0.93) reported from 26 September through 8 October 2001 at four of six sites near the WTC whose data we examined. Biomarkers were measured in a subset. Most (71%) of these women were located within eight blocks of the WTC at 0900 hr on 11 September, and 12 women were in one of the two WTC towers. Daily EIs were determined to be highest immediately after 11 September and became much lower but remained highly variable over the next 4 weeks. The weekly summary EI was associated strongly with women’s perception of air quality from week 2 to week 4 after the collapse (*p* < 0.0001). The highest levels of polycyclic aromatic hydrocarbon–deoxyribonucleic acid (PAH-DNA) adducts were seen among women whose blood was collected sooner after 11 September, but levels showed no significant associations with EI or other potential WTC exposure sources. Lead and cobalt in urine were weakly correlated with ∑EI, but not among samples collected closest to 11 September. Plasma OC levels were low. The median polychlorinated biphenyl level (sum of congeners 118, 138, 153, 180) was 84 ng/g lipid and had a nonsignificant positive association with ∑EI (*p* > 0.05). 1,2,3,4,6,7,8-Heptachlorodibenzodioxin levels (median, 30 pg/g lipid) were similar to levels reported in WTC-exposed firefighters but were not associated with EI. This report indicates intense bystander exposure after the WTC collapse and provides information about nonoccupational exposures among a vulnerable population of pregnant women.

Depending on the World Trade Center (WTC) source characteristics and the date after 11 September 2001, emissions from the WTC site during September 2001 through early 2002 affected lower Manhattan as well as immediately adjacent areas in Brooklyn and New Jersey. Environmental and personal air monitoring, dust analyses, and biomonitoring have identified that dust and air particulates and fibers, alkaline dust, and polycyclic aromatic hydrocarbons (PAHs) were greatly elevated in the first days after 11 September, with some contaminants remaining above background for 1–3 months (Lioy et al. 2002, in press; McGee et al. 2003; Swartz et al. 2003). Higher atmospheric levels, compared with contemporaneous or pre-11 September measures in mid-Manhattan, have been found for a number of chemicals, including volatile hydrocarbons (benzene, xylene, tetrachloro-ethylene), metals [lead (Pb), antimony (Sb)], and semi- and nonvolatile chlorinated hydrocarbons [e.g., polychlorinated dibenzofurans (PCDF)] (Lioy et al. 2002; Offenberg et al. 2003). Individual exposures have been assessed among firefighters and construction workers who were working close to the site on and after 11 September (Edelman et al. 2003). However, bystander exposures have not been described, although adverse health effects have been reported (Trout et al. 2002). Air-monitoring data for both particulate mass (e.g., Figure 1) and PAHs (Landrigan et al. 2004; Thurston et al. 2003) showed elevations within the first month after 11 September, with intermittent excursions after that time ascribed to meterologic conditions as well as operational changes at the site (Dalton 2003).

We have characterized and estimated WTC-related exposures among 187 women who were pregnant and were at or near the WTC on 11 September or within the subsequent 3 weeks. A preliminary report on birth outcomes in this group has been published (Berkowitz et al. 2003b).

## Materials and Methods

In 2002, we established a prospective epidemiologic study of 187 women who were pregnant and located within or near the WTC on or about 11 September. The great majority of the women recruited to this study were self-referred on the basis of extensive media publicity surrounding our investigation. Additional participants were located by sending letters to nearly 3,000 obstetricians in the greater New York City area, distributing flyers in lower Manhattan, and advertising in local newspapers. Eligibility criteria were pregnancy on 11 September 2001 or shortly thereafter and presence in one of five “exposure” zones at or near the WTC at 0900 hr (*n* = 170) on that day or within the next 3 weeks (*n* = 17). The five exposure zones were *a*) the area including and surrounding the WTC site bordered by Murray Street (north), Nassau Street (east), the Battery (south), and the Hudson River (west); *b*) the area of Manhattan south of Chambers Street excluding zone 1; *c*) south of Canal Street and north of Chambers Street; *d*) Brooklyn Heights; and *e*) the easternmost part of New Jersey across the Hudson River from the WTC (Figure 2). The women were evenly distributed (28–34%) for trimester of pregnancy on 11 September.

We used questionnaires and a time–activity log to help characterize exposures from 11 September through 8 October 2001. The questionnaire included items on socio-demographic characteristics; medical and pregnancy history; employment; potential home, work, and food exposures to PAHs (i.e., grilled foods and cigarette smoke); evacuation on 11 September through the dust and debris; perception of air quality (PAQ) after 11 September; and presence of WTC dust in the home. The time–activity log recorded all the time spent indoors and outdoors at any distinct street address in one of five zones around the WTC for 4 weeks after 11 September (Figure 2). These zones were defined by five different areas at increasing distances from WTC, which represented likely high exposures to emissions and dust depending on the location and intensity of the WTC plume (emissions) and dust impact areas. The time spent outside any of the five zones was not recorded but could be computed by subtraction. In separate analyses, no differences in exposure patterns were seen between indoors and outdoors, so we used total time spent in the exposure analyses. The time–activity logs were geocoded using Arc GIS software (ESRI, Inc., Redlands, CA) based on each street address and daily time spent within these zones.

Beginning in February 2002, we obtained blood and urine specimens from 178 women, 55 of whom were in the third trimester of pregnancy (the remainder had delivered), to determine levels of PAH-DNA adducts and other biomarkers of exposure. For 160 samples, DNA was successfully obtained from mononuclear cells by standard RNase and proteinase K treatment and phenol/chloroform extraction. Mononuclear cells have higher adducts than do granulocytes (Jahnke et al. 1990; Savela and Hemminki 1991), and thus this method should allow detection of adducts in women who were most heavily exposed to WTC fire emissions or dust. The half-life of PAH-DNA adducts in total white blood cells has been reported to be 3–5 months (Mooney et al. 1995), and the half-life of adducts in mononuclear cells should be no shorter than in total white blood cells. PAH-DNA adducts were measured by a competitive ELISA with chemiluminescence end-point detection (Divi et al. 2002). Results were the average of triplicate measurements. For 7% of samples with sufficient DNA, samples were reassayed. Repeat analysis of three positive controls resulted in a coefficient of variation of 22%. Samples with inhibition < 20% were considered nondetectable. The limit of detection (LOD) was approximately 40 adducts per million nucleotides (40 apmn), based on 20% inhibition and 10 μg DNA assayed per well. PAH-DNA adducts were categorized as below the LOD, < 60 apmn (median of detectable adducts), ≥60 apmn, and > 100 apmn. This upper category was derived from a plot showing a marked shift in the cumulative distribution at this level (> 92%).

In a randomly selected subset of 100 women, the Centers for Disease Control and Prevention (CDC) determined that these women had metals in urine and blood and organochlorines [OCs; included 40 polychlorinated biphenyls (PCBs), 7 polychlorinated dibenzodioxins (PCDDs), 10 PCDFs, and 8 polybrominated diphenylethers (PBDEs) in plasma. Random selection was done by assigning a random number obtained using the SAS function (SAS Institute, Inc., Cary, NC) to all 187 women, sorting on the random number, and choosing the first 100 women. Metals were measured in whole blood and urine using published methods (Date and Gray 1989; Guo and Baasner 1993; Nixon et al. 1999; Paschal et al. 1998). OCs and PBDEs were analyzed using high-resolution gas chromatography/isotope-dilution high-resolution mass spectrometry (Patterson et al. 1991; Sjödin et al. 2004). Serum total lipids were calculated using the values for triglycerides and cholesterol assayed with an enzymatic method (Akins et al. 1989). Urinary creatinine was assayed using a kit from Sigma (St. Louis, MO). The median was 0.43 g/L, and the maximum 1.9 g/L; two values were < 0.01 g/L. Urine metals are presented uncorrected to allow comparison with previously reported data, but adjustment for creatinine was done in multivariate models by including it as a covariate.

Limits of detection for each biomarker are given in each table in “Results.” For metals and PBDE, laboratory values were censored at the LOD. In parametric analyses, we substituted the value LOD/√2 for unreported metal levels. Because we focused on OCs with levels above the LOD in > 50% of the samples, univariate analyses of OC concentrations [four PCBs (∑PCB4), two PCDDs, and two PCDFs] changed very little whether LOD values were assigned a censored value (LOD/√2) or the actual value was reported. However, the measures of central tendency for concentrations of ∑PCB4/total PCBs were influenced by choice of surrogate value for the LOD. Few samples had ∑PCB4 congeners below the LOD, but many samples had values below the LOD for other PCBs. As a result, the ∑PCB4: total PCB ratio decreased artificially when a surrogate value (LOD/√2) was used. The mean ∑PCB4: total PCB ratio increased by 40% and the median by 50% when using LOD/√2 rather than zero or the actual value below the LOD. Therefore, we used the actual values (which were all positive) or the lowest positive value (to avoid zeroes) of congeners for the sum of PCBs (see discussion and citations in Berkowitz et al. 2003a; Fitzgerald et al. 2004). We could not use this approach for PBDEs because the lowest positive value was usually higher than the lowest reported LOD value. Therefore, because individual LOD values were given, we used the median LOD of all samples and substituted the median LOD/√2 for LOD samples in parametric analyses.

A daily dust exposure index (EI) was estimated for each woman. The EI each day was derived by reconstruction of the post-11 September WTC plume. The Computational Chemodynamics Laboratory (a research unit of the Exposure Measurement and Assessment Division of the Environmental and Occupational Health Sciences Institute, University of Medicine and Dentistry New Jersey) developed simulations of the plume that was generated by the collapse of the WTC buildings and the subsequent fires at the site (Huber et al. 2004).

The plume reconstruction was obtained by generating a set of simulations that were the result of employing the Regional Atmospheric Modeling System (RAMS)/Hybrid Particle and Concentration Transport (HYPACT) model, which is employed as part of the Modeling Environment for Total Risk (MENTOR) developed by the Environmental and Occupational Health Sciences Institute. The RAMS/HYPACT model was used to reconstruct the atmospheric dispersion of “generic” particulate matter (PM) emitted from the location of the WTC. This simulation employed a triple-nested modeling domain of 4 × 4 km (grid 1), 1 × 1 km (grid 2), and 250 × 250 m (grid 3) resolutions. The RAMS/HYPACT results were averaged over time to produce 8-hr concentrations for five zones across lower Manhattan, Brooklyn, and New Jersey. A source decay factor was incorporated into each 8-hr concentration average estimate, corresponding to a sigmoidal weakening function. This was done to simulate the decrease in the intensity of the fires and other emission sources during the days after 11 September.

The RAMS/HYPACT model accounts for advection, convection, and dispersion of emissions after the collapse of the towers, but is not constructed to model the initial wave of dust and debris. A geographic information system (GIS)-based database developed by the U.S. Environmental Protection Agency [EPA; U.S. Geological Survey (USGS) 2001] from overflight photos and satellite images provides approximate boundaries of dust/debris, allowing characterization of the extent of the area of deposition from the collapse of the WTC buildings (U.S. EPA 2002; USGS 2001). The GIS data were superimposed on the zones of concern to determine the relative impact of the dust and debris wave for use in the development of the EI for 11 September. The available images show that the dust/debris field completely covered zones 1 and 2 from 11 through 13 September 2001. During this same time period, zone 3 was also heavily covered to an area within 300 feet of zone 2 boundaries. Most remaining suspended dust was settled and/or washed away after a rain event on 14 September. For zone 1, zone 2 and the first 300 feet within zone 3 (nearest the zone 2 boundary), the airborne concentrations from resuspended dust/debris on 11 through 13 September were estimated to be approximately equal to two-thirds of the maximum plume concentration. In the remainder of zone 3, this estimate was one-third of the maximum plume concentration (in each case, the maximum plume concentration is the maximum of the 8-hr average). These weighting factors were derived from visual inspection/interpretation of the available images of the WTC plume and debris and the relative intensity of plume and dust.

Reflecting the RAMS/HYPACT model start time of 0900 Eastern Daylight Savings Time, only two 8-hr averages exist for 11 September. Therefore, the total number of hours per person was set to be ≤16 for 11 September. Because the time–activity diary for an individual does not include the specific hours spent in a particular zone in a particular day, the data sets cannot be used to directly estimate exposure based on the time of day. As a result, the daily average plume density in each zone was combined with the time–activity diary entries (i.e., time spent in each zone) to develop an average EI.

The EI accounts for time spent both indoors and outdoors. Various indoor/outdoor (I/O) concentration penetration factors have been reported and used for microenvironmental models. These values typically range from 0.05 to 0.9 (Burke et al. 2001; Thornburg et al. 2001; Vette et al. 2001). To account for the type of building ventilation system (residential/nonresidential) and a particle size range, an I:O ratio value of 0.15 was used.

The EI was calculated for 187 women for 11 September and each of the 27 days immediately thereafter. Because the exact emission amount resulting from the WTC collapse is unknown, a hypothetical unit pollutant release rate was used in the RAMS/HYPACT simulation. The simulation with a unit release provides the relative “normalized” concentration of airborne contaminants at different locations. These calculations result in a dimensionless EI. Because the values are relative, identical EIs on different days may not represent exactly equivalent daily exposures. However, the potential misclassification was small enough to justify their summation. This was shown by the correlation of the daily median EI with EPA PM monitoring data at four sites (see Results). In addition, for most women, their exposure sums were dominated by the value on 11 September or on a few other days with very high readings, because of the logarithmic nature of the EI distribution. Therefore, for the purposes of estimating total exposure, the indoor and outdoor EIs have been summed over certain time periods, with ∑EI being the 28-day (4-week) sum. If additional monitoring data become available from ongoing data analyses, the current EIs can be analyzed to determine if there is reason to improve the estimates and more accurately calibrate the EIs.

Statistical analyses were conducted using Microsoft (Redmond, WA) Excel and SAS PC (version 9; SAS Institute Inc.). The EIs were explored as continuous variables [daily values, weekly sums, and 28-day total (∑EI) for each woman] and as quantiles (dichotomized at the median). The significance of associations among categorical variables was assessed by chi-square analysis, using the Fisher exact test and the Mantel-Haenszel test for trend where appropriate. Because the exposure variables were not normally distributed, nonparametric methods (Wilcoxon rank-sum test and Kruskal-Wallis test) were used to test for differences in the medians between groups. Spearman correlations (*r*_S_) were used to examine associations between continuous variables. In addition, we used multivariate analyses to predict biomarker levels of those analytes that demonstrated any association with ∑EI [Pb, mercury, octachlorodibenzodioxin (OCDD), ∑PCB4]. For these models, the log-transformed biomarker level was the dependent variable, and the independent variables were EI (dichotomized below or above the median ∑EI) and factors that might potentially affect selected biomarkers (age, body mass index, pregnancy at blood draw, breast-feeding, smoking, race, and fish intake). We used the general linear models program (PROC GLM) in SAS with the LSMEANS option to determine whether the biomarker level differed significantly by ∑EI (below vs. above the median).

## Results

Within this cohort of pregnant women, most participants were located north and southeast of the WTC in zones 1–3 at 0900 hr on 11 September (Figure 2). Most women (133 of 187, 71%) were located within eight blocks (zones 1 and 2), including 12 women who were in a WTC tower or the complex underneath. The locations of participants at 0900 hr on 11 September were 40.6% in zone 1, 30.5% in zone 2, 17.6% in zone 3, 1.6% in zone 4, 0.5% in zone 5, and 9.1% who were not there but who reentered the area within the succeeding weeks.

Regardless of their exact location at 0900 hr on 11 September, most women either lived or worked in zones 1–5 before 11 September (33 women both lived and worked there, 38 lived there, and 113 worked there; 3 neither worked nor lived there). Therefore, although we do not have data on average time spent in these zones before 11 September, it is likely to be much higher than the durations reported in the 4 weeks after 11 September. As shown in Figure 3A, women reported spending little time in zones 1–3 immediately after 11 September, with time spent gradually increasing thereafter, with notable dips on the weekends. During the first week after 11 September, 37% of women spent some time in zones 1–3, and the percentage rose over the next 3 weeks (42, 61, and 70%). During the month after 11 September, women spent more time in zones 2 and 3 than in zone 1, which was quite inaccessible at this time. The average duration in zone 3 was 1.2 hr/day in the week immediately after 11 September, rising gradually to 3.3 hr/day for the third work week (Monday through Friday) after 11 September (mean hours per day by zone are shown in Figure 3A); time spent in zones 1 and 2 was similar in later weeks but lower than in zone 3 in the early weeks. Over the same period, average time in zone 1 was 0.2 hr/day in week 1 and 1.2 hr/day in week 3. Patterns of time spent in zones 1–5 did not differ materially with regard to whether women lived and/or worked in lower Manhattan.

In contrast to the increasing time spent in zones 1–3, the relative dust concentrations within the five zones declined rapidly during the 28 days after 11 September (Figure 3B). However, marked fluctuations in the dust and emission levels existed, consistent with changing construction, traffic, and weather patterns over this period (Dalton 2003; Landrigan et al. 2004). The individual EIs in Figure 3C were computed by combining individual-level data of the type shown in Figures 3A and B. The daily EIs exhibit a steep decline during the 3 days after 11 September, lower values during the weekends after that, and a slight shift toward increasing levels (but well below those of the first 3 days) in weeks 3 and 4, consistent with the reported time spent in zones close to the WTC in Figure 3A. The daily relative dust levels in zone 1 (Figure 3B) were correlated with PM concentrations at four stations close to the WTC that monitored PM after 22 September 2001 (Columbia University 2003) [Chambers Street: PM ≤10 μm in aerodynamic diameter (PM_10_), *r* = 0.91; PM ≤2.5 μm in aerodynamic diameter (PM_2.5_), *r* = 0.93; 290 West Broadway: PM_10_, *r* = 0.73; West Broadway–Park Place: PM_2.5_, *r* = 0.68; *n* = 7–15 per site]. Using the models of reported PM data tested for performance against daily EI (Figure 3B), we estimated the daily median PM_2.5_ or PM_10_ levels in zone 1 by extrapolation to have been > 1,000 μg/m^3^ on 11 September and > 100 μg/m^3^ from 12 September though 16 September 2001 in some locations (data not shown).

Sociodemographic factors did not differ by the ∑EI (28-day total) among participants (Table 1). The cohort has a mean age of 35 years and is largely white, married, highly educated, and nonsmoking. Women who both worked and lived in lower Manhattan on 11 September (*n* = 33) had a higher ∑EI than did women who only worked (*n* = 113), only lived (*n* = 38), or spent time for other reasons in one of the zones (*n* = 3) (Table 1). Answers to exposure-related questions about when and where women lived were strongly correlated with ∑EI, as expected from the congruity of these questions with data in the time–activity log (selected exposures are presented in Table 1). Notably, evacuation through the immediate area on 11 September led to a higher ∑EI. The median day’s EI for 11 September was 3.2 among 85 women who evacuated through the WTC debris; the 11 September EI was higher among women who remained in the debris for ≥40 min (median EI, 3.7 on 11 September; *n* = 40) than among those who stayed for 1–39 min (median EI, 2.6 for 11 September; *n* = 45) or not at all (median EI, 1.3; *n* = 102; *p* < 0.0001). Women who wore dust masks during cleaning also had higher EIs, although the reported presence of dust in the home was not associated with EI.

The pattern of EI versus time as shown in Figure 3C is explored in relation to PAQ in Table 2. Here, in week 1, the 7-day EI sum was not related to PAQ, and this was probably because few women reported spending much time in the area during week 1 (39 of 185, 21%). During weeks 2–4 after 11 September, a strong association was observed between PAQ and weekly EI sum, because women who worked or lived in lower Manhattan returned to the area (Table 2). The association between PAQ and ∑EI existed for all women in weeks 2–4 and among just those women who reported spending most of their time in zones 1–5 (i.e., 49% in week 2, 69% in week 3, 77% in week 4; *n* = 39, 92, 127, and 143, respectively).

PAH-DNA adducts were measured in 160 women. Most were nondetectable (88 of 160, 55%); the median of detectable values was 60 apmn. Eleven women had levels higher than 100 apmn, and nine of these women had blood drawn in February or March 2002 (Table 3), a time interval within the reported clearance times of PAH-DNA adducts from lymphocytes (Mooney et al. 1995). Samples collected in February/March had a significantly greater number of detectable PAH adducts (46 apmn; 64%) and a higher median value (46.7 apmn) compared with later samples (26 apmn, 30%; median, 20.0 apmn; *p* < 0.0001; Table 3). There were no consistent associations between PAH adducts and EI, when considered overall or by various temporal windows of blood draw and EI weekly sum. Neither ∑EI nor PAH adducts were associated with dietary intake of foods that may contain PAHs (e.g., broiled meat) or smoking, questions included in the questionnaire for this purpose.

Thirteen urinary metals as well as Pb, cadmium, and Hg in blood were measured in a subset of 100 women. After comparing the percentage detected and the median values of the urinary levels in our data with data from earlier reports (CDC 2003; Edelman et al. 2003), in Table 4 we present findings on urinary Sb, Cd, Pb, and uranium and blood Pb and Cd, for which levels were reported among firefighters exposed at the WTC (Edelman et al. 2003). We also included urinary cobalt because levels were weakly correlated with ∑EI. We included blood Hg because levels in the women were higher than in the National Health and Examination Study (NHANES) data and because Hg was reported as a possible WTC site contaminant (Edelman et al. 2003). None of the other urinary metals was associated with ∑EI, either among all women or among the February–March biospecimen collections. Moreover, the observed correlations with ∑EI, which were significant among 100 urine samples (*r*_S_ = 0.20 for Co and 0.21 for Pb; *p* < 0.05), were not significant among the February–March biospecimens (*r*_S_ = 0.13 and 0.12, respectively; *p* > 0.3; *n* = 44). The median values of four urine metal concentrations in Table 4 were lower than those reported in WTC-exposed or control firefighters (urinary Co was not reported). The medians of Co, Cd, and uranium were lower than among females in the NHANES data (CDC 2003), whereas urinary Pb was higher (Table 4). Blood Cd was significantly correlated with ∑EI (*r*_S_ = 0.29, *p* = 0.01), but the comparison with ∑EI above and below the median was not significant for any of these (Kruskal-Wallis test). No significant associations of blood metals with exposure were seen among the February–March biospecimens.

Selected organohalogen compounds (40 PCBs, 7 PCDDs, 10 PCDFs, 8 PBDEs) were measured in blood plasma in the same randomly selected subset of 100 women as were the metals. Data on specific PCBs, PCDFs PCDDs, and PBDEs presented in Table 5 were chosen because they were detected in > 50% of our samples, they were reported in WTC debris (PCBs, pentachlorodibenzofuran, PBDEs; Litten et al. 2003; Offenberg et al. 2003), or they were elevated in serum among firefighters (Edelman et al. 2003). PCB and OCDD levels were not significantly higher among women whose ∑EIs were above the median, and none of the other OCs or PBDEs differed significantly with respect to median ∑EI. In comparison with other reports, the median heptachlorodibenzodioxin level (30 pg/g) was similar to the adjusted geometric mean reported in exposed firefighters (28 pg/g lipid; Edelman et al. 2003). Heptachlorodibenzofuran levels were higher in exposed fire-fighters, but no levels were reported. 2,3,4,7,8-Pentachlorodibenzofuran was below the LOD in 59% of our sample. Two other OCs were detectable in > 50% of the women, including 1,2,3,6,7,8-hexachlorodibenzodioxin (median, 22 pg/g) and OCDD (median, 224 pg/g); levels were not reported for the firefighters. There were no significant associations between PBDEs and ∑EI.

We performed multivariate analyses to evaluate the association of analytes that showed some pattern of association with ∑EI, by computing the geometric means of the biomarker (dichotomized above vs. below the ∑EI median) adjusted for age, nonwhite race, being currently pregnant, breast-feeding, body mass index, and smoking. We also adjusted models for PCBs and Hg for fish intake. We found no significant differences in the adjusted geometric means with regard to dichotomized ∑EI for PCBs, OCDDs, PBDEs, Pb (blood and urine), Cd, or Hg among all 100 women tested or among the 44 women who donated blood samples in February–March 2002.

## Discussion

**Figure 1:**
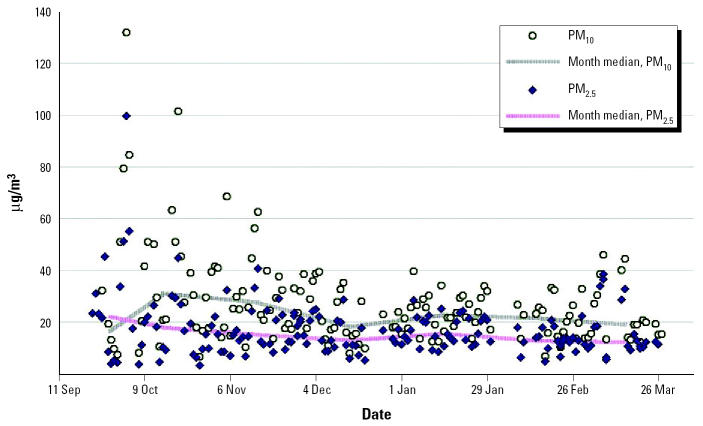
U.S. EPA particulate air monitoring approximately six blocks northeast of WTC (290 Broadway) from 25 September through 26 March 2002. PM_10_, *n* = 169 days; PM_2.5_, *n* = 181 days. Data from Columbia University (2003) and U.S. EPA (2003).

**Figure 2:**
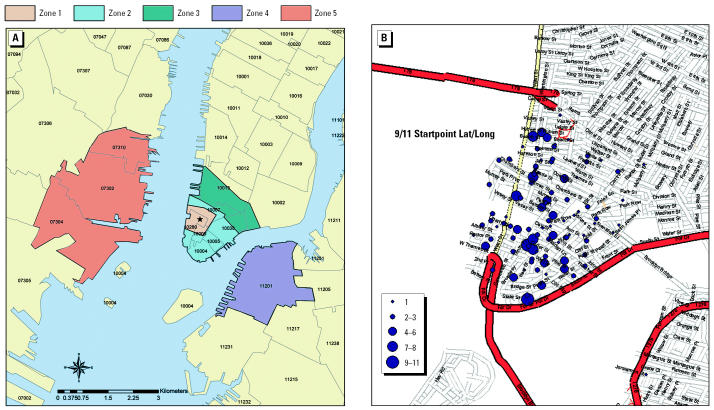
(*A*) WTC diary study zones over ZIP codes. The five exposure zones are described in “Materials and Methods.” (*B*) Start point for 166 women study participants who were in zones 1–3 at 0900 hr on 11 September 2001. WTC is the blank trapezoid just south of Vesey Street.

The predominant air pollutant at the WTC was dust composed of large-size particles (> 10 μm) (Lioy et al. 2002). Air monitoring conducted in the vicinity of the WTC after 25 September 2001 revealed elevated levels of PM_10_ and PM_2.5_ as close as six blocks northeast of the site (Figure 1). Daily PM_2.5_ levels measured five blocks east of the site from 14 September through 16 October 2001 were 15–60 μg/m^3^ (Landrigan et al. 2004). At the end of September 2001, when more air monitoring had commenced, median levels of PM_2.5_ were > 60 μg/m^3^ (one to two blocks away). At Chambers Street (six blocks north-northwest), the median PM_10_ level was 43 μg/m^3^ and the median PM_2.5_ level was 10 μg/m^3^ in October 2001. At 290 Broadway (six blocks northeast), the median PM_2.5_ level was 22 μg/m^3^ in late September (*n* = 4 days) and 15 μg/m^3^ in October, whereas the median PM_10_ levels were 32 and 30 μg/m^3^, respectively, during those time periods. However, daily excursions of PM_10_ and PM_2.5_ levels near or higher than 100 μg/m^3^ occurred periodically, depending on site operations, weather, and traffic (Columbia University 2003; Dalton 2003; Vette et al. 2001). Some distance away at Canal Street (15 blocks or about 0.7 miles north), levels were lower (median PM_2.5_, 14.3 μg/m^3^ in September, 12.5 μg/m^3^ in October 2001). At Public School 64 (about 50 blocks northeast), PM_2.5_ levels were 11.2 μg/m^3^ in September and 13.8 μg/m^3^ in October 2001 (median), which is similar to annual data at Public School 64 (13.5 μg/m^3^ in 2001, 12.1 μg/m^3^ in 2002). Women in our cohort in zone 1 were probably exposed to > 100 μg/m^3^ of particulates on 11 through 12 September, based on our extrapolation of levels of PM_2.5_ and PM_10_ from the daily EI correlations with the air monitoring data. The U.S. EPA 24-hr standard is 65 μg/m^3^ for PM_2.5_ and 150 μg/m^3^ for PM_10_ (U.S. EPA 1997).

**Figure 3:**
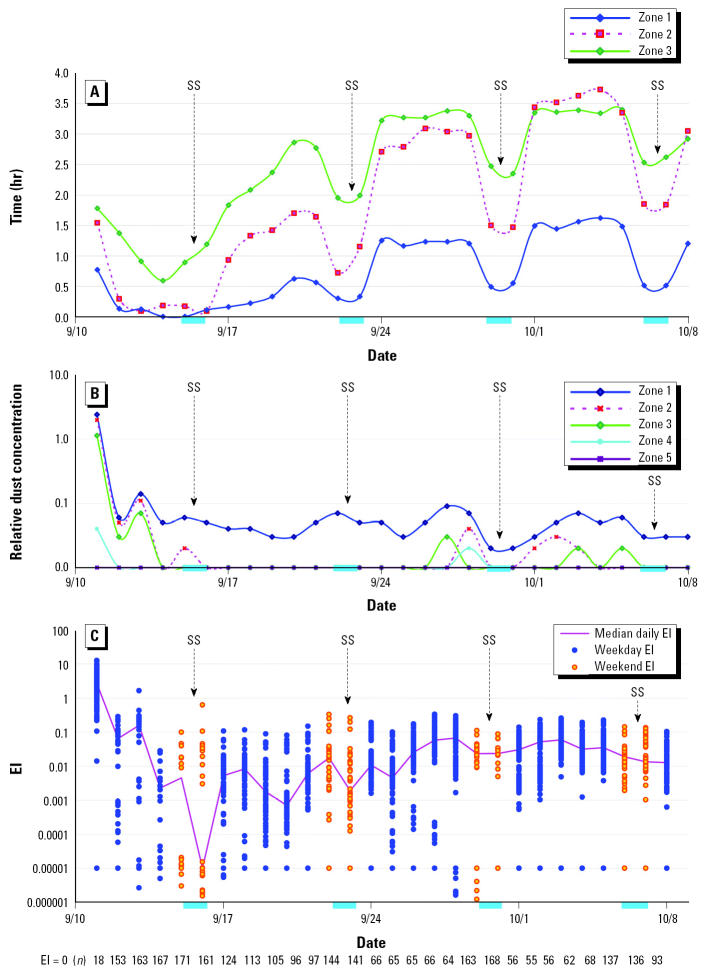
Duration and levels of exposure among 187 women participants from 11 September 2001 through 9 October 2001. SS, Saturday and Sunday; weekend dates were 15–16, 22–23, and 29–30 September and 6–7 October 2001. (*A*) Average time spent by women in zones 1–3 (indoor and outdoor combined). Average time spent in zones 4–5 (not shown) was < 0.7 hr/day on any day. (*B*) Reconstructed relative intensity of dust exposure in each zone for the same time period, derived from plume reconstruction. Values less than 0.01 were considered below the limit of reliable estimate. (*C*) Individual daily EI values for all women (*n* = 187). EIs for each woman are individual values computed from relative intensity (*B*) and time spent at all street addresses within zones 1–5 over this time period (*A*). The solid line in *C* connects the median of nonzero EI values for each day. The numbers at the bottom of *C* are the *n* values of women whose EI was zero on this day.

As the dust exposures in lower Manhattan gradually diminished in weeks 1–4 after 11 September, women gradually returned to that area for longer time periods. Therefore, fluctuation in the daily EIs (Figure 3) reflects personal activity patterns (which increased) as well as well-known changes in pollution sources and pollutant levels in lower Manhattan during this period (Dalton 2003). Consistent with the temporal decline in particulate levels, the median daily EI in our population decreased by 10- to 100-fold over the first few days (Figure 3C). After 14 September 2001, the median of nonzero reconstructed daily relative dust levels remained < 0.01 (the nominal detection limit) (Figure 3B). The significant association of EI with questionnaire data on exposure and air quality indicates that personal recollection may be a reasonable way to estimate a person’s relative exposure intensity in lower Manhattan after 11 September. For the events of 11 September, these results are reasonable because the magnitude of the changes in pollutant levels by day and by geographic location was large. Therefore, individual exposure to WTC contaminants can be ranked by assessing perceived air pollution as well as information from time–activity logs and GIS-based exposure modeling.

Uncertainties exist in our exposure estimates, including the recall data and the plume reconstruction. We used recall information to derive the GIS time–activity variables and to obtain data on PAQ from the exposed women. Plume reconstruction was used to calculate the GIS dust exposure levels. There were no air monitoring data during the first 24 hr after the events of 11 September, and the plume dust level estimates for the GIS were evaluated for precision during that time using plume density derived from satellite photographs.

The highest levels of PAH-DNA adducts were found in women whose blood was collected closest to the date of 11 September, but no association was found with ∑EI. PAH exposure around the WTC is known to have been significant (Landrigan et al. 2004). Extremely high levels of PAHs were found in settled dust (Lioy et al. 2002; Offenberg et al. 2003), and estimated air levels were > 10 ng/m^3^ in the PM_2.5_ fraction soon after the incident compared with < 1 in normal urban air and in the plume later on (Pleil et al. 2004).

Our data for each woman’s EI and zone locations were available for the day of the event through 8 October 2001, the time when peak exposures occurred around the WTC. PM and PAH levels were highest during the first 36 hr after 11 September (Offenberg et al. 2003). However, airborne PM and PAH levels were elevated for some time after 11 September (Pleil et al. 2004; Swartz et al. 2003). Therefore, PM and PAH exposures were greater in weeks 3 and 4 than in week 2, because women spent more time in the affected area as time went on. This pattern may reflect seasonality of PAH emissions, as recent New York City personal air monitoring data suggest (Perera et al. 2004). However, other studies that have found higher PAH levels in winter observed marked differences only in highly polluted areas (Perera et al. 1992; Topinka et al. 2000). With urban or tobacco smoke exposures, the PAH-DNA adducts were only slightly higher in winter than in summer (Georgiadis et al. 2001).

Metals were reported to be elevated at the WTC site debris by some but not all investigators. Blood Pb and urine Sb were higher in exposed than in control firefighters, whereas urine Cd was higher in more heavily exposed than in less heavily exposed workers (Edelman et al. 2003). When adjusted for potential confounders, we found no significant associations between metal biomarkers and EI or timing of the blood draw closer to 11 September. Metal levels other than blood Hg in these women were not higher than among firefighters or among females in NHANES. Moreover, the adjusted geometric mean for blood Hg was identical to that in the NHANES females (0.9 μg/L; Table 4) and in women of reproductive age in NHANES (1.0 μg/L; Schober et al. 2003). Although these comparisons are helpful in understanding our exposure data, they should be interpreted with caution because the three groups are very different demographically and geographically.

Organochlorines were abundant at the WTC site and in runoff from the site (Lioy et al. 2002; Litten et al. 2003; Offenberg et al. 2003), and the compounds found at the highest levels in environmental samples were those consistent with combustion products. Similarly, elevated levels of heptachlorodibenzodioxin and heptachlorodibenzofuran were reported in the most highly exposed firefighters examined (Edelman et al. 2003). In our study group, there were no significant associations between OCs and EI. The PCB levels are quite similar to levels reported in several recent studies, especially considering the differences in the populations and the laboratories performing the analyses. For example, our median ∑PCB4 level (83 ng/g lipid) is lower than that in a New York City cohort of pregnant women who were younger (median, 151 ng/g lipid; mean age, 25 years; Wolff et al. 2005). Another northern New York cohort had similar levels [geometric mean, 1.2 μg/L; estimated to be 150 ng/g lipid based on 8 g/L plasma lipids in pregnant women (Longnecker et al. 2003); mean age, 26 years; Fitzgerald et al. 2004]. Finally, levels are similar to those found in two breast milk pools from California and North Carolina, collected in 2002–2003 from mothers approximately 30 years of age (Wang and Needham 2003).

Levels of 2,3,4,7,8-pentachlorodibenzofuran were unusually high in the WTC outfall (Litten et al. 2003); this compound was not detectable in 59% of our women, although it has usually been found in breast milk at levels equal or higher than those of 1,2,3,6,7,8-hexachlorodibenzodioxin or 1,2,3,4,6,7,8-heptachlorodibenzodioxin/furan (Focant et al. 2002; Glynn et al. 2001; Yang et al. 2002). Other detected dioxins and dibenzofurans found by us (1,2,3,6,7,8-hexachlorodibenzodioxin, 1,2,3,4,6,7,8-heptachlorodibenzodioxin/furan, and OCDD) are also widely detected and are the highest detected congeners in breast milk (Focant et al. 2002; Glynn et al. 2001; Schaum et al. 2003; Schecter et al. 2002). Their relative proportions (i.e., OCDD > 1,2,3,4,6,7,8-heptachlorodibenzodioxin > 1,2,3,6,7,8-hexachlorodibenzodioxin) are similar to those among the WTC women in our cohort, even where levels were much higher (Yang et al. 2002). Furthermore, the proportions are similar to those reported from California and North Carolina 30-year-old mothers (Wang and Needham 2003), although median levels in our mothers were higher (e.g., OCDD from California, 42 pg/g lipid; from North Carolina, 84.6 pg/g lipid). This would not be attributable to age or other factors, given the effect sizes in our multivariate models (data not shown). PBDEs in our samples were somewhat lower than those reported in two milk pools (Wang and Needham 2004), but the proportions were very similar.

Our data show only a weak association of OCs with WTC-derived exposure in the month after 11 September. Levels are similar to those in firefighters with demonstrated elevations, and levels are higher than those reported in recent national data (Centers for Disease Control and Prevention 2003). Therefore, some of these compounds might have been absorbed by bystanders and firefighters in lower Manhattan after 11 September. However, the levels might not be high enough to show relationships with exposure because the WTC increment over existing body burden is not detectable. For example, if the native body burden is 20 pg/g lipid, a pregnant woman would have 600 ng total in 30 kg of adipose and circulating lipid. To see an increase in level, an absorption of 120 ng would be required, based on 20% precision in the measurements (laboratory and population variation). Our population was in varying stages of pregnancy and after delivery at time of blood collection, which greatly complicates interpretation of biomarker data, including metals and persistent organic compounds.

In summary, we were able to successfully incorporate results of a WTC plume reconstruction model into predicted exposures and doses of airborne emissions among these women who were near the WTC immediately after 11 September. The results provided daily estimates of exposure by using both geographic dust concentrations and individual activity patterns to predict individual EIs among 187 women for 28 days. Women experienced very high dust exposures; as a result of being close to the WTC in September 2001, the PM exposures were likely > 100 μg/m^3^ for women exposed immediately after 11 September. Higher PAH-DNA adducts were found in blood samples collected closer to 11 September, and personal PAQ was associated with relative dust levels estimated from plume reconstruction (EI).

## Correction

The values listed in “Materials and Methods” for the sample (165 women, 33 of whom were in the third trimester) were incorrect in the online version. Also, in Table 3, the values in the “Total” row appeared under the wrong columns. These have all been corrected here.

## Figures and Tables

**Table 1:** Selected demographic factors and activity patterns among 187 participants in the Mount Sinai WTC pregnancy study by ∑EI derived from plume reconstruction data.

	∑EI <median (*n*)	∑EI ≥ median (*n*)	Total (*n*)	*p*-Value
Interview age (years)				
< 25	1	1	2	
25–29	19	8	27	
30–34	30	43	73	
35–39	34	28	62	
≥40	9	14	23	0.12*^a^*
Race				
White	70	64	134	
Black or African American	9	11	20	
Asian	2	6	8	
Hispanic	5	8	13	
Other (mixed)	7	5	12	0.48
Marital status				
Married	83	83	166	
Living with the baby’s father	7	7	14	
Never married/separated/divorced	3	4	7	0.93
Education				
Some high school/high school	4	5	9	
Some college	12	12	24	
Bachelor’s degree (grades 13–16)	31	27	58	
Some graduate school (grades ≥17)	4	5	9	
Master’s degree	25	23	48	
Doctoral degree (e.g., JD, MD, PhD)	17	22	39	0.33*^a^*
Smoking at time of blood draw				
No	87	90	177	
Yes	6	4	10	0.50
Worked or lived in lower Manhattan before 11 September				
Worked near WTC	59	54	113	
Lived near WTC	22	16	38	
Lived and worked near WTC	9	24	33	0.019*
Neither worked nor lived near WTC	3	0	3	0.009**
Evacuation through debris on 11 September				
Did not evacuate through debris	66	36	102	
1–39 min	20	25	45	
≥40 min	7	33	40	< 0.0001
Dust in home?				
Yes	25	28	53	
No	7	10	17	0.67
Dust removal method				
Wet cloth	4	4	8	
Both wet cloth and wet mop	14	20	34	
Don’t know	7	5	12	
None of the above	2	3	5	0.76
Did you wear a dust mask while cleaning?				
Yes	10	24	34	
No	21	11	32	0.003

The median ∑EI for 187 women was 2.66 (relative dust exposure). *p*-Values are for chi-square.

a Test for trend.

* *p* = 0.019; chi-square test of the three categories of women who worked or lived near the WTC.

** *p* = 0.009; all four categories, using Fisher’s exact test.

**Table 2:** EI (dust intensity) in relation to PAQ score in lower Manhattan, by week after 11 September.

	Week 1 (EI median = 2.45)				Week 2 (EI median = 0.016)				Week 3 (EI median = 0.13)				Week 4 (EI median = 0.11)			
PAQ score in lower Manhattan (PAQ range, 1–8; *n* = 185)	No. < median	No. ≥ median	% ≥ median	Total	No. < median	No. ≥ median	% ≥ median	Total	No. < median	No. ≥ median	% ≥ median	Total	No. < median	No. ≥ median median	% ≥	Total
Women who spent most of their time in zones 1–5 (*n* = 185)
Very hazy/smoky to dense/visible haze/smoke with bad smell during some/most of the time (PAQ 3–4)	12	16	57	28	10	62	86	72	25	64	72	89	28	56	67	84
None to rarely visibly hazy or smoky with none to occasional bad smell (PAQ 1–2)	3	8	73	11	7	13	65	20	15	23	61	38	24	35	59	59
Those who did not spend most of the week in zones 1–5 (PAQ not applicable)	77	69	47	146	75	18	19	93	52	6	10	58	40	2	5	42
*p*-Value*^a^*	0.19				< 0.0001				< 0.0001				< 0.0001			
	Week 1 (EI median = 2.45)				Week 2 (EI median = 0.016)				Week 3 (EI median = 0.13)				Week 4 (EI median = 0.11)			
Women who spent most of their time in zones 1–5 during the given week after 11 September
No.	39				92				127				143			
Mean PAQ score [sum of week’s air (mean ± SD) quality at work and/or home]	3.3 ± 1.5				3.4 ± 1.2				3.1 ± 1.1				2.9 ± 1.2			
Mean ∑EI for week (mean ± SD)	3.92 ± 3.39				0.086 ± 0.15				0.22 ± 0.20				0.22 ± 0.20			
Spearman correlation of ∑EI with PAQ score (*r*_S_)	0.074, *p* = 0.66				0.288, *p* = 0.005				0.219, *p* = 0.013				0.308, *p* = 0.0002			
Women who worked or lived in zones 1–5 on 11 September including those who did not spend much time therein the 4 weeks after 11 September (*n*)
No.	185				185				185				185			
∑EI for week (mean ± SD)	2.87 ± 2.54				0.051 ± 0.115				0.16 ± 0.19				0.18 ± 0.20			
Median	2.45				0.016				0.13				0.11			

Two women who did not live or work were not present in zones 1–5 on 11 September were excluded from these analyses for this table.

a *p*-Values are for chi-square and were identical for the Mantel-Haenszel (trend test). In the 4-week analyses for PAQ versus weekly EI, the *p*-values for the two groups with PAQ values 1–2 and 3–4 (2 × 2 tables) were 0.37 (*n* = 39), 0.031 (*n* = 92), 0.21 (*n* = 127), and 0.37 (*n* = 143).

**Table 3:** PAH-DNA adducts among 160 mothers in the Mount Sinai WTC pregnancy study.

		Biospecimen collection times		Specimens collected Feb–March*^a^*	
All mothers					
PAH-DNA (apmn)	No. (%)	April–October 2002 No. (%)	Feb–March 2002 No. (%)	< Median ∑EI No. (%)	≥ Median ∑EI No. (%)
ND	88 (55)	62 (70)	26 (36)	11 (15)	15 (21)
< 60	36 (22)	14 (16)	22 (31)	14 (20)	8 (11)
60–100	25 (16)	10 (11)	15 (22)	6 (8)	9 (12)
> 100	11 (7)	2 (2)	9 (11)	5 (7)	4 (6)
Total	160	88*	72*	36	36

ND, not detected.

a By ∑EI (median = 3.30).

* Distributions are significantly different, chi-square *p* < 0.001.

**Table 4:** Blood and urinary biomarkers for metals among 100 mothers in the Mount Sinai WTC pregnancy study.

			February–March 2002 biospecimens						
	All mothers tested (*n* = 100)		(*n* = 44)		(*n* = 44)*^a^*		WTC exposed firefighters*^b^*		
	Median	% ≥ LOD	Median	% ≥ LOD	< Median ∑EI	≥ Median ∑EI	Control	Exposed	NHANES*^c^* median (females)
Urinary metals (μg/L)*^d^*									
Pb	0.82	94	0.78	98	0.75	0.89	1.0	1.2	0.60
Co	0.32	96	0.38	100	0.35	0.40	NS		0.41
Cd	0.22	98	0.20	98	0.24	0.19	0.38*^e^*	0.32	0.34
Sb	< LOD	27	< LOD	34	< LOD	< LOD	0.16*	0.20*	0.12
U	< LOD	53	0.0055	52	0.0055	< LOD	0.0075*^f^*	0.0061	0.006
Blood metals (μg/L)*^g^*									
Pb	17	100	16	100	16	16	19*	28*	13
Cd	0.30	71	0.30	55	0.30	0.22			0.3
Hg (total)	3.2	100	2.2	100	2.4	2.2	“Not higher”		0.9

Abbreviations: GM, geometric mean; NS, not significant; U, uranium. Metals were tested in a random subset of 100 women, of whom 44 gave specimens from February through March 2002; the remainder were sampled after this time.

a Median < or ≥ median ∑EI (3.62); metals were not significantly different in this analysis for values < versus ≥ EI with all or with 44 samples, both median tests (Kruskal-Wallis) and trend (Mantel-Haenszel). There were 22 samples each in the groups with values < versus ≥ median ∑EI.

b Edelman et al. 2003; GM adjusted.

c CDC 2003.

d LODs for metals in urine were as follows (μg/L): Pb, 0.3; Co, 0.08; Sb, 0.07; Cd, 0.06; U, 0.005.

e Urinary Cd was higher in more heavily exposed than in less heavily exposed firefighters, although all-exposed firefighters were not higher than control.

f Urine uranium levels were higher in more heavily exposed than in less heavily exposed firefighters, although this difference was not statistically significant.

g LODs for metals in blood were as follows (μg/L): Pb, 3; Cd 0.2; total Hg, 0.2. Metals were not significantly different in this analysis for values < versus ≥ EI with all 100 or with 44 samples collected in February through March 2002, both median tests (Kruskal-Wallis) and trend.

* *p*-Value < 0.05.

**Table 5:** Plasma OC and PBDE levels among 100 mothers in the Mount Sinai WTC pregnancy study.

	All mothers (*n* = 100)		February–March 2002 biospecimens (*n* = 44)		By ∑EI < or ≥ median (2.66) (*n* = 100)		WTC exposed firefighters*^a^*		
	Median	% ≥ LOD	Median	% ≥ LOD	< Median ∑EI	≥ Median ∑EI	Control	Exposed	NHANES*^b^* median (females)
Serum OCs*^c^*									
∑PCB4 (ng/g lipid)*^d^*	84	94	82	93	81	92	—*^e^*		< LOD
2,3,4,7,8-PentaCDF (pg/g lipid)	LOD (4.0)*^f^*	41	< LOD	50	< LOD	< LOD			< LOD (4.8)
1,2,3,6,7,8-HexaCDD*^g^* (pg/g lipid)	22	80	22	89	21	22			< LOD (7.5)
1,2,3,4,6,7,8-HeptaCDD*^h^* (pg/g lipid; *n* = 99)	30	97	24	95	31	29	19*	28*	< LOD (24.7)
1,2,3,4,6,7,8-HeptaCDF*^i^* (pg/g lipid)	7.2	79	6.2	84	7.6	6.7	—*^j^		< LOD (5.2)
OCDD*^k^* (pg/g lipid)	224	95	214	98	206	236			< LOD (145)
Serum PBDEs									
BDE-28*^l^* (ng/g lipid)	0.65	59	0.38	50	0.72	0.55			
BDE-47*^m^* (ng/g lipid)	9.7	96	8.7	93	9.1	10.2			
BDE-99*^n^* (ng/g lipid)	1.5	70	1.1	61	1.5	1.5			
BDE-100*^o^* (ng/g lipid)	1.8	96	1.5	95	1.5	1.9			
BDE-153*^p^* (ng/g lipid)	1.8	98	2.1	100	1.7	2.2			

Abbreviations: GM, geometric mean. Organochlorines were tested in a random subset of 100 women, of whom 44 gave specimens in February through March 2002; the remainder were sampled thereafter.

a Edelman et al. 2003; GM adjusted.

b CDC 2003.

c OC and PBDE levels did not differ significantly by date of collection or by ∑EI. *p*-Values (Kruskal-Wallis) were all > 0.2 by median ∑EI; the Spearman correlation coefficients were not statistically significant (*p* > 0.05). Multivariate analyses predicting the OC/PBDE levels by the median ∑EI adjusting for covariates did not change the significance of the associations. There were 50 samples each in the groups with values < versus ≥ median ∑EI.

d Median of ∑PCB4 without lipid-correction was 0.46 μg/kg. LOD for ∑PCB4 was 29 ng/g lipid.

e Not different from controls; values not reported by Edelman et al. (2003).

f Median of all LOD values; range of LODs was 1.8–11 pg/g.

g LOD, 6.2 pg/g lipid (median of LODs for all such individuals; range, 3.1–18 pg/g).

h LOD, 7.2 pg/g; range, 3.9–20.

i LOD, 4.9 pg/g; range, 2.6–14.

j 1,2,3,4,6,7,8-HeptaCDF in the firefighter study (Edelman et al. 2003) was more prevalent among all-exposed versus controls (rates not given here); adjusted odds ratio, 3.5; 95% confidence interval, 1.4–9.0 for exposed (Edelman et al. 2003).

k LOD, 78 pg/g; range, 36–240.

l LOD, 0.4 ng/g lipid (median of LODs and limits of quantitation for all such individuals; range, 0.3–0.7 ng/g lipid).

m LOD, 2.0 ng/g; range, 1–4.

n LOD, 1.0 ng/g; range, 0.4–3.

o LOD, 0.5 ng/g; range, 0.5–6 (*n* = 3).

p LOD, 0.4 ng/g; range, 0.3–5 (*n* = 2).

* *p*-Value < 0.05.
